# A social prescribing model for tackling the health and social inequalities of people living with severe mental illness: a protocol paper

**DOI:** 10.1186/s12889-025-24075-3

**Published:** 2025-09-30

**Authors:** Gerard Leavey, Rebecca Watterson, Grainne McAnee, Stephen Shannon, Kyle Boyd, Bryn Lloyd-Evans, Helen Killaspy, Ian Miller, Pamela Whitaker, Saul Golden, Gavin Boyd, Lewis Ross, Ruth Curran, Sarah Wylie, Gavin Breslin, Gavin Davidson

**Affiliations:** 1 Bamford Centre for Mental Health and Wellbeing, Ulster University, Belfast-Coleraine, UK; 2 School of Sport, Faculty of Life and Health Sciences. Magee Campus, Ulster University, Londonderry, UK; 3 Belfast School of Art, Ulster University, Belfast, UK; 4 Research Department of Epidemiology and Applied Clinical Research, Division of Psychiatry, UCL. University College London, Maple House, 149 Tottenham Court Road, London, W1T 7NF UK; 5 School of History, Ulster University, Coleraine, UK; 6 Belfast School of Architecture and the Built Environment, Ulster University, Belfast, UK; 7 Research & Lived Experience in Stigma and Mental Health (Realism): Experts by Experience research group, Bamford Centre for Mental Health and Wellbeing, Ulster University, Belfast-Coleraine, UK; 8 Inspire Wellbeing, Belfast, UK; 9 Strategic Investment Board, The Kelvin, 17-25 College Square East, Belfast, BT1 6DE UK; 10 School of Psychology, Queen’s University Belfast, Belfast, UK; 11 School of Social Sciences, Education and Social Work, Queen’s University Belfast, Belfast, UK

**Keywords:** Social prescribing, Severe mental illness, Social exclusion, Community assets

## Abstract

**Introduction:**

Health care systems have failed to address the poor physical health outcomes of people living with severe mental illness. Interventions that focus on specific health behaviours and/or lack a co-design basis show little promise. There is a need for whole systems approaches that tackle the complex issues, including social isolation, discrimination, stigma, and low motivation, that influence poor health in this population. A social prescribing model that accommodates the needs and preferences may be a way forward.

**Methods:**

A mixed methods approach that assesses the CHOICE model (Challenging Health Outcomes Integrating Care Environments) in relation to (a) the social exclusion, loneliness and social support of a cohort of people living in the community; (b) participants’ experience of social prescribing and potential improvements to the intervention; (c) understanding the implementation factors, mechanisms and outcomes; (d) the engagement and sustainment of community partnerships; (e) institutional changes in policy and practice.

**Discussion:**

Codesigned and community-based participatory interventions may be crucial in tackling the health and social inequalities experienced by people with severe mental illness. However, given the complexity of such interventions, the social prescribing model that we describe in this paper, requires considerable implementation data prior to a full trial.

## Background

Severe mental illness (SMI) is an umbrella term commonly describing conditions such as schizophrenia, schizoaffective disorders, bipolar disorder; an extended definition includes people living with a major depressive disorder or personality disorder [1]. Importantly, SMI denotes a chronic condition with profoundly negative health and social impacts. Thus, people with severe mental illness (SMI) die prematurely, up to 25 years younger than the general population, due to modifiable medical risk factors. High rates of physical multimorbidity are well-documented [2–4]. In addition to the significant harms through smoking, obesity, alcohol and substance misuse, there are health risks from weight gain related to psychotropic medication. While smoking rates among people with a mental illness are three times higher than among the general population [5, 6] they also experience greater barriers to quitting [7].

These behavioural risk factors may be better understood in the context of stigma and social exclusion. Thus, poor physical health outcomes are associated with institutional discrimination and public prejudice, which reinforce the internalized and anticipated stigma that people living with mental illness experience, generally accompanied by low self-esteem [8–10] and further disengagement from community life [11]. Moreover, people with SMI are more likely than the general population to live in disadvantaged neighbourhoods and experience high levels of unemployment, poverty, housing instability, and crime victimization [12, 13]. In relation to these factors, SMI is also associated with chronic loneliness, itself a predictor of poor physical and mental health.

While recent UK policy (*Choosing health: making healthy choices easier)* [14] sets out key principles to help the public make informed choices about lifestyles, there is a lack of evidence on the development of effective interventions to help people with SMI. A recent Cochrane Review on health advice for people with SMI found only limited evidence that physical healthcare advice alone can improve health-related quality of life [15]. Although the high risk of multimorbidity and mortality in this population has been documented for over twenty years, it is clear that much more innovative and focused work is needed in this area [16]; poor physical health outcomes and decreased life expectancy in this population have not improved [17]. Some behavioural interventions that directly attempt to influence diet, exercise, cigarette and substance misuse appear to have some success but have limitations in their longer-term implementation [18, 19]. Moreover, a recent Lancet review [17] suggested that while simultaneously considering multiple lifestyle factors may be more appropriate in the management of risk factors transdiagnostic, multifactorial approaches are not evident in the literature, but rather they focus on specific factors for individual disorders. Qualitative research with lived experience of mental illness suggests that successful interventions will require a combined or holistic approach in which mental and physical health is considered together [20]. Intrapersonal interventions targeted at specific health behaviours and beliefs may have limited impact because they fail to address important contextual barriers to health improvement. Addressing the complex and multilevel factors related to social exclusion and stigma may be crucial in the pathway to reducing health inequalities.

At the institutional level, other health-related disabilities have come to be acknowledged in policy, processes and public information, severe mental illness is largely absent. In phase 1 of the CHOICE project, stakeholder workshops with community organisations confirmed a general ‘invisibility’ of people with SMI and low understanding about their needs. Additionally, SMI participants described experiences of public hostility and rejection to which they ascribed their sense of low self-worth and reticence to visit public venues due to the anxiety of ‘having a breakdown’ and experiencing further opprobrium. Consequently, people with SMI tend to be dependent on day centres run with restricted availability, particularly at evenings, weekends, and public holidays. While aware of these limitations in provision, voluntary sector organisations remain stuck with activities in single buildings that increasingly appear as mini-institutions that hamper social inclusion. These factors suggests that several processes must occur simultaneously for health inequalities to be addressed. First, overcoming low self-esteem and social inhibitions will require sustained emotional, material and social support. In tandem, community organisations must be assisted in creating more welcoming environments and encouraged to extend their inclusion policies and practices to meet the needs of this population.

### Social prescribing

Social prescribing may function as a bridge between social inclusion and health promotion. It is generally described as a non-medical approach to addressing rising physical and mental health problems through the referral of individuals to community organisations, places and activities, such as walking groups, arts and crafts, and volunteering [21, 22]. Social prescribing (SP) is increasingly used as a non-medical approach to more appropriately tackling the social and behavioural determinants of many mental and physical health conditions. It has been used a potentially effective intervention to tackle health problems that have an underlying social dimension such as obesity and type 2 diabetes [23]. Generally based in primary care, SP is facilitated by Linkworkers who signpost to community resources and may provide ongoing support. However, the complex needs of people with SMI are not specifically catered for within social prescribing and evidence about their use for this population is lacking. While primary care has a designated key role in the physical health of people with SMI, capacity, confidence and resources to do so, are patchy, sometimes negligible. Similarly, psychiatry and community mental health teams, predominantly focused on the management of psychiatric symptoms are under resourced. Voluntary Sector Organisations (VSO) that provide community-based mental health support are commonly regarded as less stigmatising than hospital programmes and deliver group-based services (e.g., social clubs, educational classes) may provide a viable partnership in tackling exclusion and improving health. CHOICE was funded as part of the £26 million, UK Research and Innovation (UKRI) Mobilising Community Assets to Tackle Health Inequalities investment. The various projects within the programme are managed and monitored through the Arts and Humanities Research Council.

### The choice model of social prescribing for people with severe mental illness

From our co-designed inquiry and development work in a previous stage, we developed an approach to SP that combines existing models of recovery and support for people with SMI. Based within the VSOs, the Community Navigator (CN) activities will be guided by Self-determination theory, an emerging theoretical framework applied to SP interventions, focusing on innate psychological needs for autonomy (i.e., sense of volitional behaviour and choice), competence (i.e., feelings of personal effectiveness) and social relatedness (i.e., feeling cared for and caring for others) that are essential requirements for autonomous motivation for behaviour change and well-being [24, 25]. In the context of SP interventions, community workers can offer needs-supportive guidance to encourage engagement in new behaviours in the community [26]. The activities of the CNs have been clarified elsewhere as solution-focused, person-centred and non-directive [27].

In the foundation phase of the project, the team will build and maintain an internet-based digital ‘community assets’ platform, which will host a wide range of community activities, events, educational and skills training courses, and places to visit. The CHOICE team will work with a community coalition of voluntary, community and statutory agencies to prepare community assets organisations and agencies, offering training and information on the needs of people with SMI. The Community navigators are expected to have skills and experience in working with people with SMI.

SMI participants can be referred to CHOICE through self-referral, statutory services (community mental health teams) primary care, and community-based day services within the VSO directly. Each participant will receive up to 15 h of individual support from the CNs. Additionally, where possible, we will develop a peer support network. In the initial assessment, we will map current social networks by listing the people that service users know, frequented community places, and activities that they do regularly. They then indicate on a ‘map’ consisting of concentric circles, who they consider closest to within the map. Alongside the knowledge of local community and other assets which are located on the assets platform, this will form the basis of an action plan to reconnect participants to people, places, and activities that they are realistic and achievable. Each participant will be given up to £50 to assist their plans. The goals and achievements will be monitored over the 12-month period.

### Methods

The complexity inherent in social prescribing for people with SMI highlights the challenges in applying a randomized controlled trial design to examine intervention effectiveness. Moreover, prior to undertaking an RCT much more foundational work is required to identify contextual factors and mechanisms that facilitate optimal implementation of the intervention. Therefore, a robust observational study was designed to assess the feasibility and acceptability of the CHOICE programme for people with complex and diverse needs and to provide vital preparatory information for the intervention’s *potential* effectiveness in alleviating social exclusion and to determine how it may be improved and for whom. Thus, this three-year project will build and maintain a suite of community assets.

### Aims

(1) undertake a prospective cohort study of people referred to our CHOICE programme; (2) examine the engagement and experiences of experts-by-experience participants; (3) examine the ‘real-world’ issues related to the context and mechanisms of delivering the intervention; (4) ascertain the access, needs and experiences of SMI participants in their use of community assets; (5) identify structural, cultural and psychological barriers to implementation; (6) examine the relationship between social prescribing and changes in social inclusion, loneliness and quality of life; (7) assess mechanisms of inclusion and health improvement for participants and the potential for wider social impacts such as education and employment.

### Sample

We have no evidence from which we can estimate a meaningful sample size. Moreover, we are not testing a particular hypothesis but rather gathering evidence on the implementation of a model for a population with complex needs. We have estimated that over 12-months, a sample of 300 people, approximately, will be sufficient to examine change in social exclusion and capture programme evaluation data.

### Eligibility

Participants will include anyone living in the community with a severe and enduring mental health problem, who receives psychiatric services and has capacity to give informed consent to participate.

### Objectives

The Social Inclusion Questionnaire User Experience (SinQue) is a validated measure of social inclusion. An online version has been developed for use in practice and tested in VSO community supported accommodation services [27, 28]. The SInQUE assesses five domains in which people may be socially excluded: social integration; productivity; consumption; access to services; and political engagement. It generates reports of areas where the person has said they would like to be more socially included and prompts the staff member and service user to collaboratively agree priorities for care and support. CNs will arrange an interview for each participant which will include a health and social inclusion needs assessment. This will inform (a) the CN’s planning and priorities; and (b) programme impact.

### Cohort study of choice participation

Following written informed consent participants will be interviewed at programme start and follow-up at 12 months. The assessment will be recorded on an online platform and will include the following:

1. Socio-demographic questions: age, gender, diagnosis (and date), locale, accommodation type and level of support.
2. The DeJong Gierveld Loneliness Scale [29]is a brief 6-item measuring both social and emotional loneliness.
3. The Questionnaire about the Process of Recovery (QPR) [30] is a useful tool for assisting people with SMI to set goals, evaluation of these goals and promoting recovery in routine service evaluation and research trials.
4. The Lubben Social Network Scale [31] is a highly validated measure to assess network ties, and identifies persons at increased risk for social isolation who might benefit from in-depth assessment and targeted interventions.

### Data analysis

We will examine the following issues related to engagement on the CHOICE project:

(1) Participant sociodemographic and clinical characteristics (e.g. age, diagnosis, residence); (2) participants’ completion, adherence and drop-out from the CHOICE programme; (3) participant preferences for community assets. The primary outcome measure is the SinQue. Thus, we will examine statistically significant change in scores of participants between Time 1 (T1) and Time 2 (T2) based on intention to treat. During the study, we will have collected follow-up data at 12 months or as soon as they decide to no longer participate. Thus, we will examine data for all participants accepted onto the CHOICE programme regardless of completion (or partial completion). Following cleaning of the data, we will consider how to deal with missingness in scores (e.g., replace with mean, imputation). We will provide descriptive statistics of scores at Time 1 and follow up (means, medians) and then create a change variable by computing the difference in scores between T1 and T2 using parametric or non-parametric (e.g. Wilcoxon signed-rank test) as appropriate after testing for normality. Additionally, we will identify predictors or covariates associated with changes in scores and examine subgroup differences in score changes using linear regression models to do so. Tin addition to socio-demographic variables, the anticipated covariates to be included in the modelling will include diagnosis, length of illness and complex needs (e.g. multiple long-term conditions). We will follow a similar analysis for the Secondary outcomes (De Jong Gierveld Loneliness Scale [29]; Lubben social network scale [31]) using logistic regression modelling where appropriate (i.e. where measures are dichotomised). All analyses will be presented with the appropriate test statistics, p-values, and effect sizes, and Odds Ratios with 95% Confidence Intervals.

### Implementation evaluation

The Consolidated Framework for Implementation Research (CFIR) comprising a comprehensive range of constructs and five major domains, will help determine factors that influence implementation and implementation effectiveness. We will use participatory approaches to examine the CHOICE model regarding: (1) the current and emergent barriers and facilitators to achieving implementation; (2) the capacity-building and sustainability issues for the VSO; (3) flexibility of assets and sensitivity to individual personhood in addressing different levels of need; (4) interagency relationships: communication and collaboration; (5) changing perspectives on roles and responsibilities.

Throughout the project, we will use longitudinal qualitative methods to examine multiple organisational perspectives. Thus, we will undertake individual and group interviews with key stakeholders across the relevant health and social care sectors (Community, statutory). These will be done at six-month intervals (or ad hoc) to document external and contextual changes (e.g., structural, political, economic) that may impact the programme. Additionally, we seek to understand the process and impact of the programme within the community mental health services (e.g., cultural change, staff capacity and ways of working) and among community assets organisations (policy change, attitudes, beliefs). For example, what impact, if any, has the project on public and institutional stigmatisation? How does the model alter the role of the VSOs and community organisations – can they become stronger change influencers? We will also explore behaviours and policies that emerge during the programme such as (a) data sharing considerations; (b) extent of intersectoral cooperation; (c) new collaborations and synergies such as emerging connections between arts and green spaces, or the ability of the model to support people into work (paid and voluntary), training or education?

### Experts by experience perspectives

In this strand we seek to obtain the views and experiences of participants in the programme. Following enrolment on CHOICE, 20–30 participants will be recruited, based on sociodemographic and other characteristics (e.g., age, sex, ethnicity, locale, social network, loneliness, diagnosis, length of illness). We will use creative approaches at the beginning and near the 12-month participation of the project, to obtain a rich understanding of the facilitators and barriers to social inclusion and what additional changes are required for the programme. We will explore issues on self-determination, participant autonomy and personhood, the extent to which the programme was aligned facilitated personal and social development and independence. Additionally, we will explore the impact of CHOICE on the different dimensions of stigma (e.g. public, self-stigma, and anticipated) and changes in social exclusion. Did the service users experience any changes in perceptions, beliefs, attitudes, and behaviours – anticipated and unanticipated? Participants who may drop out of the programme will be asked their reasons for doing so and what might have helped them remain.

Participants will be offered a choice between *Body-mapping* [32, 33] and *River of Life* [34]. Body mapping enables participants to express and symbolise emotions, represent stories about their lived experiences through visual representations, and create an image depicting embodied experience at the intersection of multiple forms of marginalisation. It combines scientific inquiry with creativity, making it effective to explore complex ideas and experiences which can be difficult to articulate through words. The body maps produced through the participant’s CHOICE journey can be compared and analysed to determine emotional and psychological changes in areas such as self-stigma, self-image etc., as well as their experiences in CHOICE. In the *River of Life*, participants use the drawing of a river to represent the course of the CHOICE activity. The participants depict the key stages of their engagement with CHOICE through a visual map. For example, they can use images (e.g. tributaries, rough waters, rocks, flowers, fish), to illustrate positive and challenging experiences permitting discussion of the reasons behind facilitators and barriers and facilitates strategies for development and change. The findings of the research will be presented in a creative output, co-designed by the experts by experience, in the form of a zine, often used by marginalised communities as a fast and inexpensive way to spread ideas.

Participants will be supported to collectively design and create a Collective Zine that will represent their experiences of CHOICE, as well as suggestions for what they would like to change, see happen in future etc.

## Discussion

The CHOICE model is aligned with the NHS England Community Mental Health Framework [35] which seeks to remove the barriers between mental health and physical health, health, social care, voluntary, community and social enterprise (VCSE) organisations and local communities; and “to deliver integrated, personalised, place-based and well- coordinated care” across the primary-secondary care interface. Further, within healthcare provision for SMI populations, the relative goal(s) of personal recovery has shifted from one traditionally focused on reducing mental illness symptoms, to a more holistic view advocating for the adoption of valued social roles and participation, and ability to forge and maintain social relationships [36, 37].

The mechanisms by which social prescribing are thought to promote better physical health outcomes among people with SMI are seldom articulated. However, Fancourt and colleagues [38] provide a ‘multi-level leisure mechanisms framework’, which suggests that social prescribing activities (e.g. leisure, arts) activate various processes organised as biological, psychological, social and/or behavioural, leading to health and wellbeing benefits. Although popular and promising, SP operates within complex and changing professional and social environments. In this study, we have acknowledged that social prescribing for people with severe mental illness presents additional challenges that require higher levels of support sustained over longer periods of time, compared to the general population. Moreover, a wide range of needs and capacities related to sociodemographic characteristics, length and severity of illness, interests and level of social support, among many other things, must be accommodated. The anticipated heterogeneity of participant needs, preferences and experiences are explicit in the underlying theory and design of the social prescribing model, and the evaluation design. While the rationale for social prescribing generally is strong, the findings of randomised controlled trials remain unconvincing [39]. We suggest that the evaluation outlined in this paper will assist the development and implementation of improved social prescribing interventions for people with severe mental illness.

## Data Availability

No datasets were generated or analysed during the current study.
