# Correction: A social prescribing model for tackling the health and social inequalities of people living with severe mental illness: a protocol paper

**DOI:** 10.1186/s12889-025-25554-3

**Published:** 2025-12-09

**Authors:** Gerard Leavey, Rebecca Watterson, Grainne McAnee, Stephen Shannon, Kyle Boyd, Bryn Lloyd-Evans, Helen Killaspy, Ian Miller, Pamela Whitaker, Saul Golden, Gavin Boyd, Lewis Ross, Ruth Curran, Sarah Wylie, Gavin Breslin, Gavin Davidson

**Affiliations:** 1 Bamford Centre for Mental Health and Wellbeing, Ulster University, Coleraine, Belfast UK; 2 School of Sport, Faculty of Life and Health Sciences. Magee Campus, Ulster University, Londonderry, UK; 3 Belfast School of Art, Ulster University, Belfast, UK; 4 Research Department of Epidemiology and Applied Clinical Research, Division of Psychiatry, UCL. University College London, Maple House, 149 Tottenham Court Road, London, W1T 7NF UK; 5 School of History, Ulster University, Coleraine, UK; 6 Belfast School of Architecture and the Built Environment, Ulster University, Belfast, UK; 7 Research & Lived Experience in Stigma and Mental Health (Realism): Experts by Experience research group, Bamford Centre for Mental Health and Wellbeing, Ulster University, Coleraine, Belfast UK; 8 Inspire Wellbeing, Belfast, UK; 9 Strategic Investment Board, The Kelvin, 17-25 College Square East, Belfast, BT1 6DE UK; 10 School of Psychology, Queen’s University Belfast, Belfast, UK; 11 School of Social Sciences, Education and Social Work, Queen’s University Belfast, Belfast, UK

**Correction: BMC Public Health 25**,** 3211 (2025)**

**https://doi.org/10.1186/s12889-025-24075-3**

Following publication of the original article [1], the authors identified an error in Pamela Whitaker’s name and an error in the affiliation listed for both Pamela Whitaker and Saul Golden.

The incorrect author name is: Pamela Whittaker.

The correct author name is: Pamela Whitaker.

Pamela Whitaker is affiliated to Belfast School of Art, Ulster University, Belfast, UK.

Saul Golden is affiliated to Belfast School of Architecture and the Built Environment, Ulster University, Belfast, UK.

The author group has been updated above and the original article [1] has been corrected.
